# Performing Economic Evaluation of Integrated Care: Highway to Hell or Stairway to Heaven?

**DOI:** 10.5334/ijic.2472

**Published:** 2016-10-19

**Authors:** Apostolos Tsiachristas, K. Viktoria Stein, Silvia Evers, Maureen Rutten-van Mölken

**Affiliations:** Health Economics Research Centre, Nuffield Department of Population Health, University of Oxford, Oxford, GB; International Foundation for Integrated Care, Oxford, GB; Department of Health Services Research, CAPHRI – School for Public Health and Primary Care, Maastricht University, Maastricht, Netherlands; Institute of Health Policy and Management, Erasmus University Rotterdam, Rotterdam, The Netherlands

**Keywords:** health economics, economic evaluation, health technology assessment, integrated care, complex intervention

## Abstract

Health economists are increasingly interested in integrated care in order to support decision-makers to find cost-effective solutions able to tackle the threat that chronic diseases pose on population health and health and social care budgets. However, economic evaluation in integrated care is still in its early years, facing several difficulties. The aim of this paper is to describe the unique nature of integrated care as a topic for economic evaluation, explore the obstacles to perform economic evaluation, discuss methods and techniques that can be used to address them, and set the basis to develop a research agenda for health economics in integrated care. The paper joins the voices that call health economists to pay more attention to integrated care and argues that there should be no more time wasted for doing it.

## Introduction

Health economists are increasingly interested in integrated care for chronic conditions. This is because the rapidly increasing prevalence of chronic conditions reduces population’s health, increases the demand for health and social care [1]. It also has negative macroeconomic consequences for consumption (i.e. reduced demand), capital accumulation (i.e. less investments), labour productivity (i.e. less output per working hour) and labour supply (i.e. availability of human resources). [2]. Health economists support healthcare decision makers with evidence in finding an adequate response to these challenges by studying the changes in demand for healthcare, investigating the efficiency of health technologies, studying their financing mechanisms, and advocating the efficient allocation of scarce resources. The findings of health economics support decision-makers to define the right mixture of healthcare interventions to maximise the health and well-being of society as well as to meet the preferences and needs of patients.

One of these responses is the provision of integrated care. This refers to “initiatives that seek to improve outcomes for those with (complex) chronic health problems and needs by overcoming fragmentation through linkage or coordination of services of different providers along the continuum of care” [3]. It puts the patients and their individual needs and preferences in the centre and organizes care around them. Integrated care is seen as a promising means to increase productive efficiency in care for people with chronic conditions [4]. According to the triple aim framework, as advocated by the Institute for Healthcare Improvement, integrated care aims to 1) improve population health, 2) improve patient experience with care, and 3) reduce costs [5].

Economic evaluation in integrated care is still in its early years. It faces several difficulties mainly due to the fact that integrated care is a complex package of interventions with unclear definition, composition, and application, which deviates substantially from simple interventions that are traditionally subject to health economic analysis. However, the urge for a wider implementation of integrated care to address the needs of people with chronic conditions and improve efficiency calls for more evidence-based decision-making based on thorough economic evaluations. The existing evidence about the economic impact of integrated care available in the thin scientific literature is inconclusive [3]. The main reasons are the great variation in interventions, and the relatively weak methodological approaches to evaluate integrated care [6]. Many studies have called for more reliable and replicable economic evaluation of integrated care [7] and recognised that current evaluative frameworks may not be sufficient to address complex interventions [8], because these interventions require different costing methods and their outcomes extend beyond Quality Adjusted Life Years (QALYs). Therefore, a modified framework with extended costing methods and outcome metrics that include the non-health benefits (e.g. satisfaction) of integrated care may be needed.

The aim of this paper is to describe the unique nature of integrated care as a topic for economic evaluation, explore the obstacles to perform economic evaluation, discuss methods and techniques that can be used to address them, and set the basis to develop a research agenda for health economics in integrated care. The following sections are structured along the components of economic evaluations as suggested in guidelines issued by health technology assessment agencies in Europe [9].

## Integrated care defined as complex intervention

**Figure 1:**
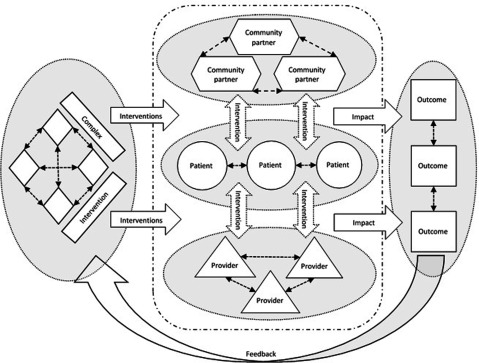
Illustration of complex intervention.

Health technologies such as medicines, diagnostic tests, medical devices, and surgical procedures are considered to be “simple” interventions because they are usually delivered by one care provider or provider organisation and the outcome is a result of the intervention and the interaction between the patient and the caregiver. Complex interventions are different. Their common characteristics include one or more of the following: a) various interacting components, b) targeting groups or organizations rather than or in addition to individuals, c) a variety of intended (and unintended) outcomes, d) they are amendable to tailoring through adaptation to the context in which they are introduced and learning by feedback loops of patient- and provider-experiences and outcomes, and e) effectiveness is impacted by behaviour of those delivering and receiving the intervention [10]. Figure 1 illustrates how a complex intervention is diffused to different groups of recipients, interacts, and impacts different outcomes. Integrated care is a good example of a complex intervention. The World Health Organization (WHO) defines it as “a concept bringing together inputs, delivery, management and organization of services related to diagnosis, treatment, care, rehabilitation and health promotion. Integration is a means to improve services in relation to access, quality, user satisfaction and efficiency” [11]. Similar definitions of integrated care can be found elsewhere [12,13]. Based on the WHO definition, integrated care may be considered an ultra-complex intervention or according to Shiell et al., 2008 a complex system [14]. This is because integrated care is composed of multiple complex interventions (e.g. computerised decision support and self-management support), it behaves in a non-linear fashion (i.e. change in output is not proportional to change in input), and the interventions interact with the context in which they are implemented. For example, the Chronic Care Model (CCM), on which many integrated care programmes have been based, provides a framework of elements that must be considered when developing improvement strategies for providing care for people with chronic conditions, originally including: (a) self-management support, (b) decision support, (c) delivery system design, (d) clinical information systems, (e) health care organization, and (f) community resources and policies [15].

## Comparator

Economic evaluation is a comparative analysis. Even if it is not possible to identify control groups, the relative efficiency of integrated care still needs to be assessed. In general, comparators used in economic evaluations frequently include active comparators such as current practice, best available alternative, or alternative levels of treatment intensity, different variations of similar programs etc. Identifying an appropriate comparator for integrated care is challenging. Standard practice, frequently called “usual care”, is often an appropriate control but it can be at least as complex as the intervention being evaluated and may change over time by national or regional policy reforms that stimulate the evolution of usual care for an individual with one or more chronic conditions towards integrated care. As a result, usual care may have become a low intensity integrated care. Comparing integrated care models that differ in terms of their intensity or comprehensiveness maybe a good alternative when appropriate control groups without integrated care are difficult to identify [16,17]. However, the room for improvement by implementing a more intense or comprehensive programme may be reduced. Hence, the competing alternatives to be considered in an economic evaluation include: a) integrated care (complex intervention) to simple interventions delivered in current clinical practice, b) integrated care to usual care (considered also as complex intervention), c) various components of integrated care to each other or the sequence in which they were introduced, or d) all the above. Although, it is not straightforward which pair of competing alternatives to choose and each option has pros and cons, evaluation guidelines suggest the evaluation of a complex health intervention accompanied by a detailed description of the components rather than disentangling the effects of the individual components [10,18]. Arguably, the interdependence of the interventions creates synergy effects. As a result the total cost-effectiveness of integrated care is not a linear summation of the partial cost-effectiveness of the interventions provided. For example, a thorough diagnostic assessment, which is not followed by a mutually agreed treatment package based on a patient’s personal goals is unlikely to be of benefit to the patient [19]. However, the benefits of the latter are likely to be greater when based on a broad assessment of impairments, symptoms, functional limitations, disease perceptions, health behaviour and quality of life.

## Study design and data

Most evaluation studies of integrated care are observational studies and very often lack a control group [6]. Besides the difficulty of creating an appropriate control group, other reasons for adopting an observational design include financial considerations, difficulties in identifying suitable participants, concerns about the generalizability of the results, and ethical considerations [20]. However, observational studies raise major concerns about the potential sources of bias and confounding factors that may jeopardize attribution of effect (or causality). Experimental designs such as randomised clinical trials (RCTs) are considered as the most robust designs to infer causality. Since integrated care includes interventions on organizational level and the risk of contamination (i.e. the control group is affected by the intervention) is high, cluster-RCTs could be considered as an adequate study design. Even in that case, experimental designs may face similar problems as observational studies in inferring causality when evaluating complex interventions such as integrated care. This is due to hidden differences in the context with which the treatment and control groups interact that may critically affect the results [21]. Standardization of interventions would be a solution to replicate the results in other settings but in the case of integrated care, it would preclude its adaptability to the local context and would treat it as a simple intervention [22]. Moreover, it is recognised that health interventions that are observed to be efficacious and cost-effective in the context of highly structured randomized trials may not be effective or cost-effective once they are made available in practice, under less controlled conditions [23].

**Figure 2:**
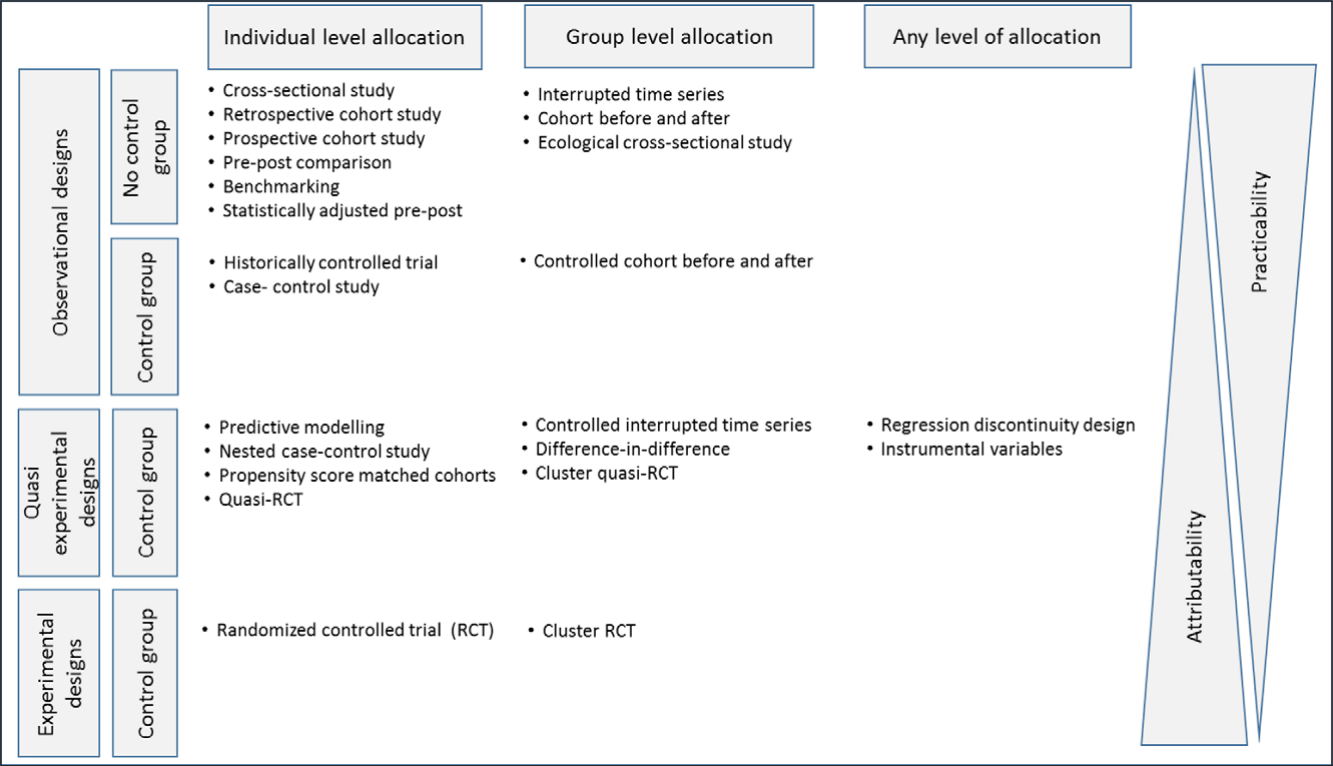
Study designs by type and level of allocation. Source: adapted from a series of RAND reports [20,26,27].

Quasi-experimental designs or natural experiments may be the best alternative when evaluating integrated care because they involve the application of experimental thinking to non-experimental situations. They widen the range of interventions beyond those that are amendable to planned experimentation and they encourage a rigorous approach to use observational data [24]. Natural experiments are applicable when control groups are identifiable and when groups are exposed to different levels of intervention. Natural experiments using regression adjustment and propensity-score matching could reduce observed confounding between the comparators while, difference-in-differences, instrumental variables, and regression discontinuity could reduce the unobserved confounding between the comparators. A combination of these techniques is also possible in the evaluation [25]. Figure 2 provides an overview of study designs to be considered in the evaluation depending on the availability of a control group and degree of experimenting.

Data availability and quality is another important factor to be considered when choosing a study design. Routine data might be of good quality and comprehensiveness but it can be costly or time consuming to access it and lengthy procedures can be applicable to merge data from different sources as confidentiality should be secured. In addition researchers have lack of control of the type of outcome measures included in the routinely collected data. In the absence or inadequacy of routine data, survey data could be used in the economic evaluation. However, the quality of survey data depends on the validity of the questionnaire, the response rate, the missing observations, and data comprehensiveness (consider that lengthy surveys with many measures lead to low response rates). Ideally, routine data would be combined with survey data in the evaluation of integrated care and would be interpreted with the support of data collected from qualitative research. However, a complete economic evaluation based on different data sources requires substantial financial and human resources. Even when resources are not an issue, lack of evaluation culture, related shortage of capacity and reluctance of payers or providers to engage in evaluation might challenge the evaluation of integrated care [28].

## Evaluation period

Most guidelines issued by health technology assessment agencies worldwide suggest to adopt a lifetime horizon in economic evaluation of medical innovations [29]. However, most evaluation studies of integrated care had an evaluation period of a year and some were extended up to 3 years [6]. This short to medium-term evaluation period may fail to capture the full effect of integrated care. This is because it takes at least 3–5 years for health management initiatives to identify “true” programme effectiveness due to lags in full implementation [30]. This may not even be long enough to study the effects of the preventive interventions in the integrated care package. However, adopting a follow-up period longer than 5 years may increase the risk of failing to attribute effects to integrated care because in the long-term, the intervention and eventually control groups are contaminated with other interventions and health policy reforms [31]. Common sense would suggest to consider the start and end points of integrated care to determine an adequate evaluation period but none of these points is clear-cut in integrated care. An exact baseline measurement for evaluation is often hard to determine because the preparation and development of some integrated care interventions may have occurred way before that point. Failing to capture these efforts would underestimate the development costs of integrated care [32]. Determining the end point of integrated care is challenging as well. Integrated care interventions may be delivered one-off (e.g. 8 sessions of self-management support) or repeatedly (e.g. monitoring of high risk patients, establishment of multi-disciplinary teams, and development of integrated ICT system). Thus, the (partial) effects of integrated care are expected to be recurrent in time.

A way of extending the evaluation period without extending the official research period, is to set up a continuous routine monitoring system that tracks a core set of outcomes over time, not as part of the research but as part of routine practice. This can guide managers, healthcare providers, and payers, and may even be used to motivate patients when they have access to their own outcome data. The challenge is to choose this core set, which eventually will have to change over time to reflect continuous improvement and changing objectives that are to stakeholders.

## Outcome measures

Integrated care, as being a complex intervention, impacts many outcomes on different levels. These outcomes could be categorised in process indicators of the organization and delivery of care, patient’s satisfaction with care, access to care, informal caregivers’ satisfaction and quality of life, patients’ lifestyle and risk factors, patients’ ability to self-manage and cope with disease, clinical outcomes, functional status, quality of life, wellbeing, and mortality [3,33,34]. Besides objective outcome measures (e.g. blood tests, smoking status, date of death) the outcomes can be measured with patient-reported outcomes measures (PROMS), patient-reported experience measures (PREMS), and patient activation measures (PAMs). These outcomes encompass the argument of Huber et al., that health should be defined more dynamically, based on the resilience or capacity to cope and maintain and restore one’s integrity, equilibrium, and sense of wellbeing [35] as well as the capabilities approach of Amartya Sen including ‘empowerment’ which can be viewed as a type of capability that measures the ‘ability of a person to function’ [36,37]. Even advocates of QALYs as measurement to support decision-making would argue that all of these outcomes cannot be captured in a single unit of measurement. Moreover, literature suggests that the QALY may not be relevant for decision-making at the level of provider organisations and insurers, when reimbursement decisions have already been made at national or regional level [38]. In that case, the decision that needs to be taken is not whether to fund integrated care but which type of programme should be provided, to whom and how in day-to-day practice. Thus, QALY is not a relevant measurement to be used in clinical decision support systems, which are primarily informed by changes in clinical outcomes, health risk factors, care processes, and behaviour. Multiple outcome measures, measured at multiple levels (e.g. patient, GP practice, and community) and eventually from different perspectives (e.g. providers and patient) should be employed to assess whether the triple aim of integrated care has been reached. However, the measurement burden, especially for frontline clinicians, should not be underestimated.

Some of these outcome measures could be used to inform performance indicators to facilitate the provision of financial incentives for integrating care. This would go beyond the performance indicators currently used in pay-for-performance schemes (e.g. in England [39]) by informing integrated care specific indicators and group specific indicators (e.g. disadvantaged people or people with multi-morbidity). Examples of such measures have been issued by WHO and include for example care planning and coordination, shared decision making, and medication review in older adults [40]. Looking at the care continuum, performance indicators could be assigned with different importance in time. For example indicators of physical improvements may be more important in the short term and indicators of psychological and social improvements in the long term for a patient who had a stroke. Furthermore, absolute and relative performance indicators could be combined to stimulate high-performing providers to maintain their performance levels and motivate low-performing providers to achieve relatively high performance [41,42]. However, financial incentives linked to individual process or outcome indicators have been found to have unwanted effects like a reduction in focus on unmeasured outcomes and gaming strategies. Perhaps financial incentives linked to population-level outcomes can overcome these effects, although this involves the challenge of creating a mutual sense of shared responsibility among providers to achieve these outcomes.

## Measurement and valuation of costs

Similar to outcomes, integrated care also impacts a broad range of costs, inside and outside the health care system. As a result, the societal perspective (i.e. considering all costs at societal level) is preferred to the narrower health care perspective when estimating the costs of integrated care. A full societal perspective would include the impact of integrated care on all sectors of the society (e.g. social care, workforce, education, security and justice). However, such a perspective would demand complex, time-consuming, and costly data collection and cost calculation. Thus, health economists may want to restrict the societal perspective to include only those societal costs that are expected to be impacted by the integrated care programme under evaluation. For example, costs in the education and justice sectors might be relevant for inclusion in an economic evaluation of integrated care programmes for adolescents with mental conditions but not for a programme targeting adults with diabetes. Furthermore, integrate care programmes require substantial development costs (including but not limited to training costs, ICT costs, and costs of redesigning the care delivery process) and implementation costs (such as multidisciplinary team meetings, the costs of coordination between care-givers, the costs of monitoring and feedback). These costs are commonly carried by the organization that implements the programme and should be included in the economic evaluation.

A “minimum” set of cost categories relevant in the evaluation of integrated care may include [33,43]: 1) the development costs of integrated care, 2) the implementation costs of integrated care, including process oriented costs, 3) the costs of health and social care utilization (including long-term care), 4) the costs of informal care and 5) the costs of productivity loss due to absence from paid work or reduced productivity while at work. But again, the selection or relevant cost categories depends on the context. For example, if an already developed integrated care programme was implemented in another setting, then the development costs would not be relevant for inclusion in the analysis.

Development and implementation costs of integrated care could be collected via surveys or interviews with managers or financial controllers of integrated care programmes. A study systematically collected these costs by using a template based on the CostIt instrument of the World Health Organisation (WHO) [32,44]. This study could provide inspiration on how to treat overhead and capital costs as well as how to amortize development costs of integrated care.

Measuring and valuing all other cost categories could follow current practices and guidelines in health economic literature. The costs of health and social care utilization could be measured retrospectively by standardised questionnaires like the Client Service Receipt Inventory (CSRI) [45] or based on routine or claims data. The CSRI also includes questions for residential care, criminal justice service and state benefits. Patient travelling costs and productivity costs could also be collected via standardized surveys [46]. Developing and applying questionnaires to measure resource use customized to a study would be an alternative of using existing questionnaires but this would require additional research time to validate them [47]. Unit costs could be gathered similar to traditional economic evaluations [48]. When national average unit cost prices are not available or not precise enough, activity-based costing may be a useful alternative in estimating service costs of integrated care [49,50]. However, this approach is very costly and in many cases impractical to be performed in large scale economic evaluations [51].

## Broader economic evaluation

Considering the broad range of health and non-health outcomes for inclusion in the evaluation of integrated care, the adoption of cost-benefit analysis (CBA) -in which all benefits are expressed in monetary terms- and cost-effectiveness analysis (CEA) -in which the effects are measured in natural units (e.g. life years gained)- is precluded because these methods have a single measure of outcome [48,52]. Even if all outcomes of integrated care could be expressed in monetary terms and included in CBA [53], it would be very time-consuming and costly to do so and the objections against assigning monetary values on health would still remain [37]. Performing a cost-utility analysis (CUA), which is the most widely used evaluation method and believed to have a comprehensive outcome measure, might be problematic in the case of integrated care because as mentioned earlier, a QALY does not capture the non-health benefits of integrated care (e.g. patient satisfaction with the process of care delivery). Therefore, a cost-consequence analysis (CCA) seems an adequate alternative because it presents a range of outcomes alongside costs. CCA probably fits better with real-world decision-making, in which decisions are made based on other criteria besides cost-effectiveness but it does not support a systematic ranking of alternative interventions based on their cost-effectiveness [54]. Multi-Criteria Decision Analysis (MCDA) could overcome this limitation of CCA by supporting a systematic comparison of different alternatives based on their performance on various pre-specified criteria (i.e. a range of outcomes and costs) [54]. In this process, different criteria are weighted according to their relative importance to the decision by different stakeholders, including patients. Hence, MCDA is a sophisticated method for comparing complex interventions, such as integrated care, incorporating all relevant categories of outcomes and costs [55,56].

A framework to evaluate integrated care based on MCDA is reported in the literature [33]. The challenge for performing MCDA in this context is to determine a set of criteria relevant for decision-making and assign weights based on the preferences of stakeholders in integrated care. Whether the new composite measure that results from a MCDA can include other criteria than health and non-health benefits (e.g. costs) is debated [57,58]. If the new composite measure only includes benefits, then a new incremental cost-effectiveness ratio (ICER) threshold value for one unit of additional benefit on this composite measure may need to be determined to support reimbursement decisions. However, MCDA may also be used alongside and as a supplement to the existing deliberate process, serving to structure the discussions and feed back to decision makers the weights implicit in their decisions [59]. This may particularly apply when other criteria than benefits are included in the composite measure. Inter-sectoral costs and consequences may also be addressed by combining CCA and MCDA [60].

## Determinants of cost-effectiveness

Similar to many complex interventions, the cost-effectiveness of integrated care depends on the provided interventions and their combination. There is evidence about the (cost-) effectiveness of most interventions included in integrated care [40,61,62,63,64]. However, theoretical and conceptual studies on integrated care strongly suggest that the value of integrated care is in the combination of interventions. This is because integrated care “is not a discrete and immediately replicable intervention and its elements should be treated as a totality” [65]. Ham (2010) argues that the tenth characteristic of a high performing chronic care system is the link between individual interventions that transforms them into a coherent whole and has an additional effect [66]. It is unclear whether this effect of combining different interventions is additive or multiplicative but it surely is the synergy and interaction between interventions that contributes to the overall effect. Therefore, the evaluation of integrated care should be undertaken at an aggregated level [22]. Moreover, the complexity of integrated care in terms of intervention intensity [27] and comprehensiveness [16] as well as its uptake and successful implementation [67] may impact outcomes and costs. Especially the development and implementation costs would increase with complexity [32]. The target population is another determinant of integrated care cost-effectiveness [17]. This may largely be explained by the fact that integrated care involves behavioural aspects. Literature shows that behaviour interventions are highly cost-effective but not for everyone [68]. This notion is also shared by the National Institute for Health and Care Excellence (NICE) in England where thorough subgroup analysis is recommended when evaluating behavioural change interventions [18]. Finally, the existence of economies of scale and economies of scope may influence development and implementation costs of integrated care and therefore its cost-effectiveness.

## Policy evaluation and implementation analysis

The implementation of integrated care in many countries was supported by new forms of financing and payments [7,69,70]. This is because adequate funding and payment systems with financial incentives that steer behaviour towards collaboration between professionals are prerequisites for the successful implementation of integrated care [2,71]. Examples include the reduction in co-payments for patients participating in disease management programmes in France, the performance based payment system in England that stimulates GP adherence to clinical guidelines, the bundled payment in The Netherlands where care groups receive a single annual payment for a patient to cover the (mostly primary) care for a particular chronic disease. Positive evidence from the implementation of such financial incentives and payment schemes is reported in the literature [72,73,74,75,76].

These incentives may either be considered as behavioural interventions that are part of an integrated care programme or they may be seen as part of the local context with which the integrated care programme interacts. In the former case, a broad policy evaluation may accommodate the implementation of integrated care and accompanying payment reforms simultaneously. In the latter case, payment reforms could be seen as strategies to successfully implement integrated care. As a result, the application of Value of Implementation analysis [77,78] may be employed to provide the overall cost-effectiveness of implementing integrated care with the support of financial incentives. However, it would be hard to disentangle the impact of the payment reform from the effect of the care reform on health care expenditure and care quality.

## Standardised reporting

Reporting of methods and results should be systematised to allow traceability and transferability of the health economic evidence in integrated care. A thorough description of the interventions provided as part of integrated care, and eventually in the control group, including their timing and intensity and the involved providers should provide a clear understanding of “what” was evaluated. The methods employed and the assumptions made in the economic evaluation should also be clearly stated regarding the “how” was it evaluated and the results of subgroup analysis should highlight “for whom” it was cost-effective. Existing statements such as the CHEERS statement [79], the STROBE statement for observational studies [80], and the disease management quality assessment instrument developed by Steuten et al., [81] could be used to standardize reporting. Including a periodic evaluation and detailed documentation of the provided interventions (including the control group, if available) in the stream of integrated care interventions, could provide meaningful information about the full and sustainable cost-effectiveness of integrated care.

## Discussion and research agenda

The complexity of integrated care and the substantial resources needed to collect reliable data appears to have challenged health economists to evaluate the cost-effectiveness of integrated care to date. Economic evaluations published in health economic journals mostly focus on single elements of integrated care [82,83,84,85,86]. There is need for that to change and health economists to understand the peculiarities of integrated care as intervention under evaluation. Recently, the Journal of Health Economics issued a call for a special issue on integrated care. This is certainly a step forward. On the health services research side, health economists were not involved in many evaluation studies so far, which presumably resulted in low quality evidence on cost-effectiveness. Economic evaluations are frequently piggy back tailed in the effectiveness evaluation of integrated care but this needs to be changed because there is a clear need for better understanding and communication between health economists, researchers from other disciplines, clinicians, payers and decision-makers during the set-up of an evaluation study.

Since economic evaluation could facilitate the (re-)designing of integrated care, funding for methodological research in this field should be available to health economists [87]. International collaboration of health economists should work on the methodological challenges, exchange experience in economic evaluations, and issue guidelines for best evaluation practice. The Health Economics Special Interest Group (HE-SIG) of the International Foundation for Integrated Care (IFIC) is an example of such an initiative [88]. This paper is an initial attempt to address the challenges for a thorough economic evaluation of integrated care and provide possible solutions to overcome them (Box 1). It could become a stepping stone for future discussion of health economists in the HE-SIG and other related groups.

Box 1 Summary of the most important suggestions and points for consideration to perform economic evaluation of integrated care

- It is better to evaluate integrated care as a complete package of several interventions rather than to investigate the contribution of each single intervention in the package.
- Usual care is likely to be the most suitable comparator.
- Quasi-experimental designs or natural experiments may be the most appropriate study designs, provided that appropriate matching techniques are used when comparing integrated care with a comparator service.
- A core set of wellbeing, health, clinical and non-health outcomes should be defined and attached to performance indicators, preferably at population level.
- A combination of routine and survey data should be used to measure the indicators mentioned above.
- A continuous routine monitoring system could support a long evaluation period.
- A minimum set of costs may include the costs of development and implementation, resource utilization, informal care, and productivity loss.
- Cost-consequence analysis accompanied with Multi-Criteria Decision Analysis might be an appropriate and convenient method of economic evaluation in integrated care.
- The intervention intensity and comprehensiveness as well as its uptake and successful implementation may impact outcomes and costs.
- Economies of scale and economies of scope may influence the cost-effectiveness of integrated care.
- Financial incentives may be seen as behavioural interventions that are part of integrated care or as part of the local context.
- Standardised methods of reporting should be adopted in the evaluation of integrated care.

Similar to previous studies [8], this paper suggests to extend or use the current health economic methods correctly in integrated care, rather than to invent new ones. It joins voices that call for a broader economic evaluation of integrated care. A consensus should be reached about whether this could be achieved with the proposed method of employing a cost-consequence analysis operationalized by an application of MCDA or by adopting a more welfarist approach such as a cost-benefits analysis, which is popular among health economists for evaluating public health interventions [89]. In this discussion, policy makers should also be involved after having been presented with the pros and cons of each evaluation method. In the case where MCDA is employed in the economic evaluation, researchers should determine a core set of criteria relevant for decision making in integrated care and assign their weights from an international perspective to allow cross-national comparisons of integrated care models.

Moreover, it would be interesting if future studies would investigate differences in access to integrated care programmes by socio-economic status and region. The economic evaluations should address equity issues such as whether integrated care should be provided to everyone in need or only to those who are expected to increase its cost-effectiveness. Consensus about costing methods (e.g. when to use activity based-costing instead of existing unit costs in integrated care) has to be reached and instruments to assess the study quality in economic evaluation of integrated care similar to existing ones [81,90] have to be developed.

Special attention should be paid to multi-morbidity because it requires more complex care than the care needed to treat single chronic conditions. More complexity means more challenges for health economists to evaluate integrated care for people with multi-morbidity and more resources needed for the evaluation. Modelling the effects and costs of patients with multi-morbidity is an obstacle in performing economic evaluation because most disease progression models are disease specific and include only some concordant co-morbidities (e.g. myocardial infarct in a diabetes type two model). Development of more comprehensive disease progression models is necessary in this matter.

Several models have been developed to evaluate complex interventions [91,92,93,94]. Their common elements are the importance of using behavioural theory and mixed-methods to understand the mechanisms that drive the effectiveness of a complex intervention. From an economic evaluation perspective, this is important in determining the mechanisms that influence costs and outcomes and designing a study capable of answering the questions: which interventions, for which patients, in which settings, using which resources? As a result, these theories and methods should be further explored in the future in order to systematize their inclusion in the economic evaluation of integrated care. In addition, health economics should support policy makers in Eastern and South European countries, where action plans to integrate care have been recently published to support policy makers in taking the first serious steps towards that direction [95,96]. A thorough economic evaluation should be integral part of their implementation to inform decision making.

Beyond the scope of economic evaluation, health economists may further investigate the economic consequences of ageing, the impact of integrated care on the demand and supply of health and social care and health insurance, the efficiency of integrated care systems, methods to incorporate the results of economic evaluation in financial agreements, and the suitability and impact of current and innovative financing and payment schemes for delivering integrated care. A comprehensive contribution of health economics in paving the way towards integrated care may be the stairway to policy makers’ heaven.

## Conclusion

This paper joins the voices that call health economists to pay more attention to integrated care. The complexity of this intervention should be seen as a challenge for health economists to explore new dynamics in this research field. The solutions to the challenges described in this paper may be the basis for future research. This is the best time to expand health economics towards integrated care because the urgency to increase efficiency in care for chronic conditions is increasing rapidly. Health care decision makers need evidence on integrated care now.
